# Dietary Palygorskite-Based Antibacterial Agent Supplementation as an Alternative to Antibiotics Improves Growth Performance, Blood Parameters, and Rumen Microbiota in Sheep

**DOI:** 10.3390/antibiotics12071144

**Published:** 2023-07-02

**Authors:** Shujie Li, Yue Liu, Hanfang Zeng, Chanjian Wang, Zhaoyu Han

**Affiliations:** College of Animal Science and Technology, Nanjing Agricultural University, Nanjing 210095, China; lishujie@njau.edu.cn (S.L.); 15119418@njau.edu.cn (Y.L.); 2020205004@stu.njau.edu.cn (H.Z.); 2021805091@njau.edu.cn (C.W.)

**Keywords:** chlortetracycline, palygorskite-based antibacterial agent, diets, growth performance, rumen fermentation, rumen microbiota, sheep

## Abstract

This research aimed to investigate the effects of a palygorskite-based antibacterial agent (PAA) as an alternative to antibiotics on growth performance, blood parameters, and rumen microbiota in sheep. A total of 120 sheep were randomly divided into five groups of six replicates with four sheep each. Sheep were fed a basal diet, an antibiotic diet supplemented with 500 g/t chlortetracycline (CTC), and a basal diet supplemented with 500, 1000, and 2000 g/t PAA for 80 d, respectively. Supplementation with 2000 g/t PAA and 500 g/t CTC increased the average daily gain (ADG) of sheep compared with the control group (*p* < 0.05). Diets supplemented with 2000 g/t PAA and 500 g/t CTC reduced (*p* < 0.05) the feed:gain ratio (F/G ratio) in the overall periods. Dietary supplementation with 1000 g/t PAA significantly increased albumin and total protein (*p* < 0.05). A significant positive correlation was found between growth hormone concentration and PAA supplementation (*p* < 0.05). In addition, compared to the control group, the CTC group had higher growth hormone concentration and lower lipopolysaccharide concentration (*p* < 0.05). No difference was observed between the five groups in terms of rumen fermentation characteristics (*p* > 0.05). At the phylum level, the relative abundance of *Proteobacteria* was lower in the PAA 2000 and CTC 500 groups than in the control and PAA 500 groups (*p* < 0.05). At the genus level, a significant decrease (*p* < 0.05) in the relative abundance of RuminococcaceaeUCG-010 was observed in the PAA 1000, PAA 2000, and CTC 500 groups compared with that in the control group. In addition, the relative abundance of Prevotella1 (*p* < 0.05) was higher in the PAA 2000 group than in the control group. These findings indicate that dietary supplementation with PAA has ameliorative effects on growth performance, blood parameters, and rumen microbiota, with an optimal dosage of 2000 g/t for sheep.

## 1. Introduction

Antibiotics have long been used as animal growth promoters and have brought considerable economic benefits to livestock producers [1,2]. However, issues, such as drug-resistant bacteria, antibiotics residue, and food safety caused by the widely use of antibiotics have long attracted people’s attention [3,4]. On 1 July 2020, the Ministry of Agriculture and Rural Affairs of China announced that it was forbidden to add antibiotics to animal feed [5]. However, the prohibition of antibiotics in animal feed has resulted in poor animal growth performance and an increased incidence of intestinal and systemic diseases, ultimately resulting in significant economic losses [6]. Several studies have shown that antibiotics as feed additives can promote animal growth, reduce diseases, and augment benefits [7,8]. Therefore, it is important to identify new feed additives to replace antibiotics.

Palygorskite (Pal) is a clay mineral containing hydrous magnesium aluminum silicate with a large specific surface area. It has biological functions, such as good adsorption performance and ion exchange performance, and is non-toxic and pollution-free. Pal can adsorb toxic and harmful substances, such as intestinal pathogenic bacteria and mycotoxins, and plays a key role in the treatment of diarrhea and gastrointestinal diseases in animals [9,10]. It is mostly used as a raw feed material and additive in animal production. Most previous studies on the use of Pal in various animal have dealt with the original Pal itself and not its modified form. Studies have revealed that modified Pal has an extensive function in animal diets. Pal can be post-modified through physicochemical reactions to enhance its biological activity in reared animals owing to its good adsorption performance and strong ion-exchange capacity [11,12]. Supplementing Zn-bearing Pal to the diet can promote the growth of broilers to a level comparable to that of an antibiotic [13]. Our previous in vitro study showed that thermally modified Pal and Zn-loaded Pal can improve rumen microbial fermentation, increase bacterial diversity, and promote volatile fatty acid (VFA) formation and nitrogen utilization [14]. The results of an antibacterial test showed that palygorskite-based antibacterial agent (PAA) could influence the growth of pig-derived Escherichia coli, Salmonella [15].

In this study, a new type of PAA was prepared by loading Zinc oxide (ZnO) with strong antibacterial activity and quarternized modified chitosan onto the surface of Pal with a rod-like morphology. It not only exerts the function of Pal to adsorb harmful bacteria, but also increases the nutritional and antibacterial properties of zinc (Zn), which has multiple effects. As an alternative to antibiotics, PAA could promote growth performance and intestinal barrier function of broiler chickens [5]. However, there is limited research on the potential of PAA as an alternative to the antibiotics used in sheep. Therefore, different doses of PAA were used in the diet, and their effects on growth performance, biochemical indicators, and rumen microbiota in sheep were investigated.

## 2. Materials and Methods

### 2.1. Pal

PAA was kindly provided by Shenlite Biological Technology company (Jiangsu, China). The main components of PAA were pal/ZnO/glyceryl laurate/chitooligosaccharide (QACOS). It was produced using purified Pal as a carrier.

### 2.2. Animal Experimental Design

A total of 120 healthy Hu sheep (half males and half females) with similar body weights (15–20 kg) were selected. The sheep were randomly divided into five dietary treatments, with six replicates per treatment and four sheep per replicate. The sheep in the five treatment groups were fed the following full mixed-ration granule diets for the 80 d feeding trial: (1) basal diet (control group), (2) basal diet + 500 g/t PAA (PAA 500 group), (3) basal diet + 1000 g/t PAA (PAA 1000 group), (4) basal diet + 2000 g/t PAA (PAA 2000 group), and (5) basal diet + 500 g/t chlortetracycline (CTC 500 group). All sheep in each treatment group were reared in a house with free access to food and water, and males and females were reared in separate pens. The ingredient compositions and calculated nutrient levels are listed in Table 1.

The premix provided the following per kg of the diet: VA 16,000 IU, VD 25,000 IU, VE400 IU, Fe 35 mg, Cu 20 mg, Zn 65 mg, Mn 40 mg, I 0.2 mg, Se 0.3 mg, Co 0.15 mg.

### 2.3. Growth Performance

The initial body weight and the final body weight of the test sheep was measured. The daily feed intake and leftover feed amount for each repeated group of sheep were recorded. The daily dry matter intake and average daily gain (ADG) were calculated; the feed:gain (F/G) ratio was calculated based on the ADG and daily dry matter intake.

### 2.4. Sample Collection and Measurement

One sheep was randomly selected from each replicate for slaughter at the end of the experiment. All sheep were fasted for 02312 h and deprived of water for 2 h before slaughter. The live and carcass weights were recorded after slaughter. Blood samples (approximately 10 mL) were collected from the jugular vein using a vacuum blood collection tube before slaughter. The serum was separated by centrifugation at 2500 *g* for 20 min at 4 °C and then stored at −20 °C until analysis. The serum was used to analyze insulin, growth hormone (GH), serum total protein (TP), albumin, globulin, IGF-1, lipopolysaccharide (LPS), and urea nitrogen levels. The rumen digesta sample were collected and the pH values were determined using a portable pH meter immediately after slaughter. The rumen digesta were then strained through four layers of cheesecloth. After collection, ruminal contents were immediately divided into two parts on ice and stored in liquid nitrogen for subsequent microbiome and short-chain fatty acid analyses [16].

### 2.5. Microbiome Sequencing and Bioinformatics Analysis

The genomic DNA of the samples was extracted using a DNA extraction kit (No. 12888, QIAGEN, USA), and the DNA concentration was determined using agarose gel electrophoresis and NanoDrop2000 (Thermo Fisher, Wilmingto, NC, USA). Using genomic DNA as a template, PCR amplification was performed using barcode primers and Tksgflex DNA polymerase (No. R060B, Takara, Berkeley, CA, USA). For bacterial diversity analysis, the V3V4 variable regions of 16SrRNA genes were amplified with the primer pairs 343F(5′-TACGGRAGGCAGCAG−3′) and 798R(5′-AGGGTATCTAATCCT-3′).

Raw data are in the FASTQ format. The trimmomatic software Raw was used to assemble the paired-end sequences [17]. To ensure the accuracy of the results, accurate impurity removal was performed, and sequences containing ambiguous bases (ambiguous), single-base highly repetitive regions (homologous), and sequences that were too short in length were removed. This was achieved using the vsearch software (v2.3.4; VSEARCH, GitHub, San Francisco, CA, USA, https://github.com/torognes/vsearch, accessed on 20 October 2020) to split the sequence into multiple OTUs according to the sequence similarity after preprocessing the sequence data to generate high-quality sequences [18]. A sequence similarity ≥97% was classified as an Operational Taxonomic Unit (OTU). The representative sequences of each OTU were selected by QIIME software (version 1.8.0; http://qiime.org/, accessed on 20 October 2020) and then all representative sequences within the database were compared and annotated [19].

### 2.6. Statistical Analysis

The data of growth performance, rumen fermentation parameters, blood parameters were analyzed by ANOVA using SPSS (2008) statistical software (Ver. 16.0 for Windows, SPSS Inc., Chicago, IL, USA). Tukey’s test was used to examine the differences among five treatment groups. The relative abundance of rumen microbial among the five experimental groups was compared using the Kruskal–Wallis H test, and the value of *p* < 0.05 was considered statistically significant.

## 3. Results

### 3.1. Animal Growth Performance

**Figure 1:**
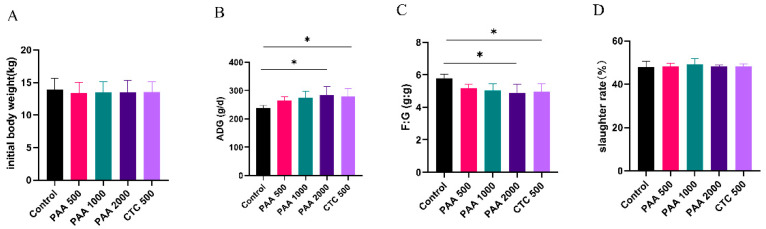
Effects of PAA supplementation on growth performance of sheep. Initial body weight (**A**); ADG, average daily gain (**B**); F:G, feed/gain ratio (**C**); slaughter rate (**D**). Different symbol at the same time differ (*, *p* < 0.05).

As shown in Figure 1, no significant differences were observed in the initial body weight. Compared to the control group, supplementation with 2000 g/t PAA and 500 g/t CTC increased the ADG of sheep (*p* < 0.05). The F/G ratio were lower in the 2000 g/t PAA group and 500 g/t CTC group than in the control group in the overall periods (*p* < 0.05). However, no significant differences (*p* >0.05) were observed in slaughter ratios among the five groups.

### 3.2. Blood Parameters

**Figure 2:**
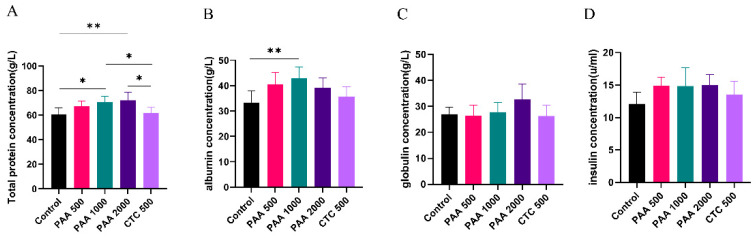
Effects of PAA supplementation on the blood parameters of sheep; (**A**) total protein concentration; (**B**) albumin concentration; (**C**) globulin concentration; (**D**) insulin concentration. Different symbol at the same time differ (*, *p* < 0.05; **, *p* < 0.01).

**Figure 3:**
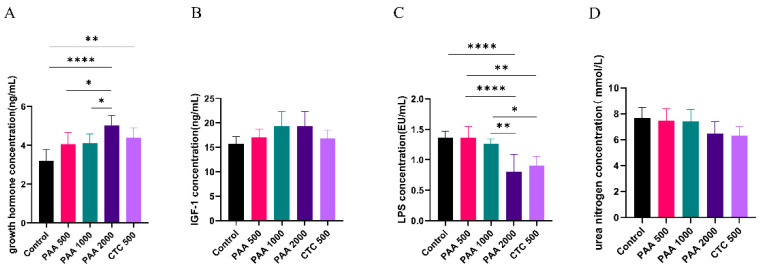
Effects of PAA supplementation on the blood parameters of sheep; (**A**) growth hormone concentration; (**B**) IGF-1 concentration; (**C**) LPS lipopolysaccharide concentration; (**D**) urea nitrogen concentration. Different symbol at the same time differ (*, *p* < 0.05; **, *p* < 0.01; ****, *p* < 0.0001).

As shown in Figure 2, the concentrations of globulin, insulin, and GH in the blood did not differ among the five groups. Dietary supplementation with 1000 g/t PAA significantly increased the albumin levels. Furthermore, dietary supplementation with 1000 g/t PAA and 2000 g/t PAA significantly increased total protein levels. As shown in Figure 3, no differences in IGF-1 or urea nitrogen concentrations were found in the PAA supplementation diets. However, a significant positive correlation was observed between growth hormone concentration and PAA supplementation. In addition, the CTC group had higher GH concentrations and lower LPS concentrations compared to the control group (*p* < 0.05).

### 3.3. Rumen Fermentation Parameters

The effects of PAA supplementation on rumen fermentation characteristics are shown in Table 2. No differences were found among the groups in terms of rumen fermentation characteristics.

### 3.4. Microbiome Structure

**Figure 4:**
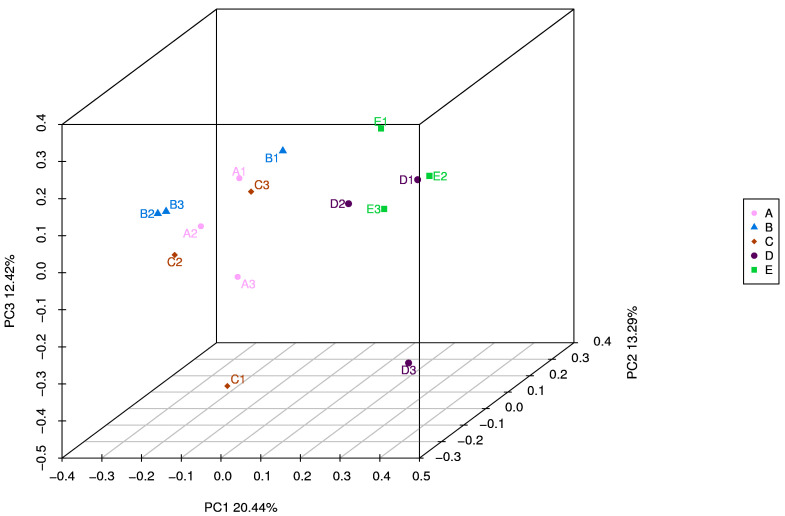
A = CON group, B = PAA 500 group, C = PAA 1000 group; D = PAA 2000 group; E = CTC 500 group; Unweighted UniFrac metric PCoA of microbial diversity in CON, PAA 500, PAA 1000, PAA 2000, and CTC 500 groups. The percentage of variation explained by PC1, PC2, PC3 are indicated on the axis.

To explore the regulatory effect of PAA in the rumen microbiota of sheep, we performed 16S RNA gene sequencing of rumen digesta samples (Table 3). The dietary PAA and CTC supplementation could affect the α-diversity indices, including the observed species Choa1, Simpson, and Shannon indices (*p* < 0.05). The number of species observed was significantly higher (*p* < 0.05) in the control and PAA 500 groups than in the PAA 2000 group. The Chao 1, Simpson, and Shannon indices were lower (*p* < 0.01) in the PAA 2000 group than in the CON, PAA 500, and PAA 1000 groups. The results of unweighted UniFrac distance-based PCoA showed that the rumen-associated bacterial communities of the CON, PAA 500, and PAA 1000 groups were gathered, while those of the PAA 2000 and CTC 500 groups were detached (Figure 4).

**Figure 5:**
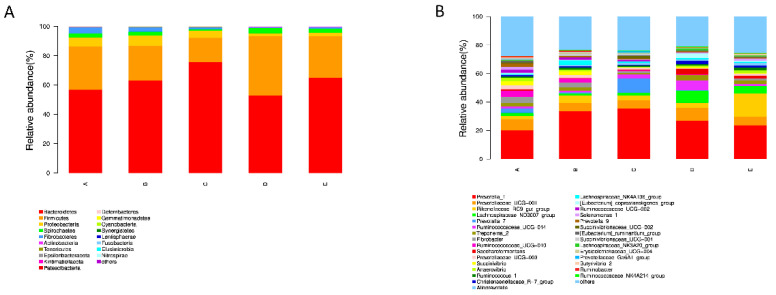
Distribution of the bacterial community composition across the five treatments. (**A**) Phylum level; (**B**) genus level.

**Figure 6:**
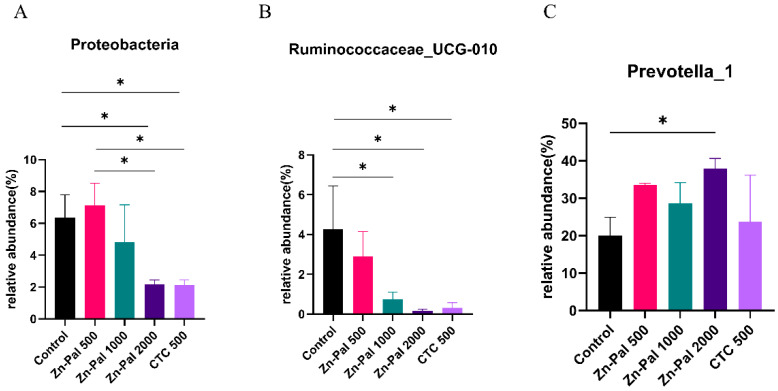
Differences in the rumen microbiota across the five treatments. (**A**) Phylum level (Proteobacteria); (**B**) genus level (Ruminococcaceae_UCG-010). (**C**) genus level (Prevotella_1). Different symbol at the same time differ (*, *p* < 0.05).

In the present study, to further investigate the effects of PAA on rumen microflora, we performed statistical analyses at different microbial taxonomic levels. At the phylum level, 18 bacterial phyla were detected in the rumen samples (Figure 5A). Among them, *Bacteroidetes* (average 62.6%), *Firmicutes* (average 27.7%), and *Proteobacteria* (average 4.5%) were the most abundant. The relative abundance of Firmicutes and Bacteroidetes was not affected by PAA and CTC supplementation. However, the relative abundance of Proteobacteria was lower in the PAA 2000 and CTC 500 groups compared to the control and PAA 500 groups (Figure 6A). At the genus level, *Prevotella_1* (average 30.1%), *Prevotellaceae_UCG001* (average 6.8%), *Rikenellaceae_RC9_gut_group* (average 6.1%), and *Lachnospiraceae_ND3007_group* (average 3.8%) were the dominant genera in the rumen microbiota. A significant decrease (*p* < 0.05) in the relative abundance of *Ruminococcaceae_UCG-010* was found in the PAA 1000, PAA 2000, and CTC 500 groups compared with the control group. In addition, the relative abundance of *Prevotella_1* (*p* < 0.05) was higher in the PAA 2000 group than in the control group.

## 4. Discussion

As China completely banned the use of antibiotics in animal feed in 2022, we need to develop new alternatives to antibiotics to maintain animal health and production. Previous research has indicated that Pal could improve animal performance [5,20,21]. Supplementing the diet of broilers with 1000 mg/kg PAA can reduce the feed conversion ratio [5]. Moreover, Zn-bearing clinoptilolite could affect the ADG of broilers from 1 to 21 days, and its supplementation could completely replace CTC to improve growth performance [22]. Pal supplementation had positive effect on weight gain and linearly increases feed intake in duck [21]. Remarkably, Pal supplementation in pig diets can improve the performance of commercial farms [23]. In the current study, compared to the control group, supplementation with 2000 g/t PAA and 500 g/t CTC increased ADG in sheep. Diets supplemented with 2000 g/t PAA and 500 g/t CTC led to a decrease (*p* < 0.05) in the F/G ratio in the overall periods. This finding is consistent with that of other investigators, who observed that diets supplemented with Pal could improve animal growth performance [13,24]. The main ingredients of PAA used in this research included Pal, QACOS, ZnO, and essential oils. It has various biological activities, including antioxidative, anti-inflammatory, antibacterial, and immunomodulatory activities, which may explain its growth-promoting effects in animals [25,26,27,28,29,30,31].

Blood is composed of plasma and blood cells. It regulates the activities of body organs, transports nutrients, and defends against foreign harmful substances [32]. Its components are affected by various factors, such as the endocrine status of the animal, nutritional level of the diet, and stage of growth and development [33]. Total protein is composed of albumin and globulin, which reflect the digestibility and utilization of protein by animals to a certain extent [34,35]. In a previous study, Ahmad et al. reported that nano-zinc oxide can significantly reduce the levels of low-density lipoprotein, total cholesterol, and triglycerides in broiler chicken serum and significantly increase the level of high-density lipoprotein [36]. Supplementation of the diet with oil/clinestone tends to reduce triglyceride concentrations, with little effect on other biochemical indicators, such as total protein and urea acid [24]. With an increase in the PAA content of animal diets, the total protein and albumin contents in the serum of weaned piglets showed an upward trend, and the ratio of white globules showed a downward trend [37]. However, dietary disaggregated Pal has no significant effect on the serum biochemical indices of broilers [38]. In the current study, dietary supplementation with 1000 g/t PAA significantly increased the albumin and total protein levels. In addition, a significant positive correlation was found between growth hormone concentration and PAA supplementation. Thus, PAA may improve protein metabolism and enhance immune function in animals. Endotoxins are cell wall components of gram-negative bacteria composed of LPS, which is the main pathogenic component in the process of inducing an inflammatory response. Excessive bacterial endotoxin content can lead to sugar and fat metabolism disorders, causing damage to the liver, kidneys, spleen, gastrointestinal tract, and other organs [39,40]. Danli et al. reported that after the injection of bacterial endotoxins in growing geese, symptoms such as lack of energy, decreased feed intake, slow growth, and decreased immunity were observed [41]. Yilong et al. showed that supplementation with modified attapulgite can significantly reduce LPS content in laying hens [42]. In addition, it was reported that attapulgite can significantly reduce serum LPS levels in weaned piglets [43]. The results of this study are consistent with these results. Adding modified Pal and aureomycin to the diet can reduce LPS content in the serum of mutton sheep. The above results are attributed to the fact that modified Pal can use its adsorption properties to absorb harmful bacteria and their metabolites in the intestinal tract.

There are several kinds of bacteria in the rumen, and various bacteria work together to degrade feed into metabolites, providing sufficient energy and nutrients for the host [44]. Thus, the rumen microbiota and rumen microbial fermentation are closely related to host productivity, health, and well-being [45]. Some studies have shown that different diets and feed additives can affect rumen fermentation and microbiota [46,47]. Previous research reported that thermally modified attapulgite and Zn-loaded attapulgite can improve rumen microbial fermentation and promote VFA formation and nitrogen utilization [14,48]. Furthermore, attapulgite contributes to the synthesis of VFAs and bacterial proteins [49]. However, this study does not support previous research in this area. In the current study, PAA supplementation had no effect on the rumen fermentation parameters. Possible reasons for this include the different animal species and amounts of attapulgite added.

The rumen is a big microbial ecosystem consistuing of various rumen microbes, including bacteria, protozoa, fungi, and viruses [45]. The diversity and richness of rumen microbes are the key factors affecting rumen function [50,51]. The present study showed that the low addition of PAA did not affect α-diversity indices, and compared to the control group, the PAA 2000 group had the lowest observed species richness, Chao1, Simpson, and Shannon indices. The results found in this study may indicate decreased species diversity and richness of sheep rumen microbiota. Previous research on the effects of Pal on the α-diversity index has yielded mixed results. Jin et al. showed that Pal supplementation increases bacterial diversity in the cecal contents of chickens, as evidenced by increases in Chao1 and the observed species [10]. Chalvatzi et al. reported that the gut microbiota is more homogeneous during laying in the Pal group than in a control group [9]. Furthermore, it was also reported that the dietary supplementation of Pal does not affect the cecal bacterial α-diversity of weaned piglets [52]. We speculate that this was due to the difference in the amount of attapulgite added and the loading of attapulgite. PCoA analysis indicated that the composition of sheep rumen microbial communities varied greatly under different treatments.

In the present study, Firmicutes and Bacteroidetes dominated the rumen microbiome of all groups, which is consistent with the results of previous studies conducted on sheep [46,53]. Firmicutes and Bacteroidetes play a major role in the metabolism, digestion, and absorption of proteins and starch [54,55]. The relative abundances of Firmicutes and Bacteroidetes were not affected by the supplementation with PAA. In addition, only one bacterium was affected by PAA supplementation at the phylum level. The relative abundance of Proteobacteria was lower in the PAA 2000 and CTC 500 groups than in the control group. Proteobacteria play a pivotal role in fermentation [56]. Proteobacteria consist mostly of pathogenic bacteria [57]. All members of the phylum Proteobacteria are gram-negative bacteria with an outer membrane composed mainly of LPS [58]. Campylobacter, Salmonella, *E. coli*, and Vibrio are the main pathogens in the phylum Proteobacteria [59]. Related studies have shown that Zn-containing Pal exhibits antibacterial activity against E. coli K88 in the artificial gastrointestinal tract of piglets [60]. Wang et al. reported that feed supplementation with zinc clinoptilolite can protect broiler performance and intestinal health from *S. pullorum* infection [61]. In this study, PAA supplementation reduced the abundance of Proteobacteria. This can be partly explained by the fact that high doses of PAA inhibit the growth of harmful bacteria. The previous results of this study also showed that the concentration of LPS in the blood was reduced, indicating that the addition of PAA could reduce the abundance of Proteobacteria. At the genus level, a significant decrease in the relative abundance of Ruminococcaceae_UCG-010 was observed in the PAA 1000, PAA 2000, and CTC 500 groups compared to that in the control group. In addition, the relative abundance of Prevotella1 (p < 0.05) was higher in the PAA 2000 group than in the control group. Ruminococcaceae_UCG-010, which belongs to the family Ruminococcaceae, has previously been shown to cause fiber degradation and butyrate production [62,63]. Moreover, Prevotella_1 belongs to the Prevotella_1 family, which reportedly possesses the ability to degrade fiber sources [64]. A previous study showed that supplementation with tea saponins could increase the relative abundance of Prevotella1, indicating that saponins can effectively enhance carbohydrate metabolism, especially during hemicellulose digestion [64]. In this study, changes in Ruminococcaceae_UCG-010 and Prevotella_1 indicated that PAA might have an effect on carbohydrate metabolism.

In conclusion, this research suggests that dietary supplementation with PAA improves growth performance and maintain animal health by reducing the concentration of LPS in serum. Also, PAA improved growth performance and regulated rumen microbiota in a fashion similar to that of antibiotics. PAA may be applied as a potential alternative to antibiotics, with an optimal level of 2000 g/t in sheep feed demonstrated by the present study.

## Figures and Tables

**Table 1:** Composition and nutrient levels of the basal diet (DM basis) %.

Ingredients	Content, % of DM	Chemical Composition, % of DM	Content, % of DM
Corn	30.8	Dry matter	87.19
Soybean meal	5.8	Metabolic energy (MJ/kg)	16.91
Peanut meal	3.0	Crude protein	14.61
Bean straw	24.0	Neutral detergent fibre	49.71
Corn germ meal	15.0	Acid detergent fibre	13.04
Rice husk	5.0	Ether extract	4.71
Wheat middling	2.0	Ash	10.06
Molasses	1.0	Ca	1.08
Malt root	6.0	P	0.62
Limestone	2.0		
Premix	5.0		
Total	100		

**Table 2:** Effects of PAA supplementation on the rumen fermentation characteristics.

Items	CON	PAA 500	PAA 1000	PAA 2000	CTC 500	SEM	*p*-Value
pH	6.43	6.53	6.46	6.43	6.44	0.02	0.67
Total VFA (mmol/L)	54.08	51.19	60.74	37.74	59.55	5.04	0.643
Acetate	39.03	34.45	37.99	29.72	36.71	2.95	0.903
Propionate	14.23	10.28	14.38	7.14	12.98	1.25	0.357
Butyrate	6.43	3.28	4.76	4.27	4.9	0.48	0.378
Valerate	0.85	0.49	0.71	0.61	0.74	0.07	0.696
Isobutyrate	1.57	1.01	1.12	1.44	1.46	0.12	0.604
Isovalerate	2.76	1.65	1.76	2.08	2.68	0.23	0.438
Acetate/propionate (A:P)	2.82	4.29	2.84	4.44	2.89	0.69	0.468

**Table 3:** Effects of PAA supplementation on the α diversity of ruminal bacterial communities.

Items	CON	PAA 500	PAA1000	PAA 2000	CTC 500	SEM	*p*-Value
Simpson	0.9816 b	0.9808 b	0.9644 b	0.9373 a	0.9660 b	0.004	0.002
Chao1	2797.4 b	2831.3 b	2628.8 b	2330.7 a	2577.4 ab	57.8	0.014
Observed species	2214.2 b	2243.8 b	2037.9 ab	1771.1 a	2005.1 ab	55.3	0.016
Shannon	7.5 b	7.4 b	6.9 b	6.4 a	7.1 b	0.12	0.005

^a–b^ Means having different superscripts in the same row differ significantly (*p* < 0.05).

## Data Availability

Data are available online in NCBI and the accession number(s) was PRJNA955016.
